# Tanshinone IIA ameliorates Aβ transendothelial transportation through SIRT1-mediated endoplasmic reticulum stress

**DOI:** 10.1186/s12967-023-03889-y

**Published:** 2023-01-20

**Authors:** Can Wan, Xiao-Qi Liu, Mei Chen, Hui-Han Ma, Guang-Liang Wu, Li-Jun Qiao, Ye-Feng Cai, Shi-Jie Zhang

**Affiliations:** 1 Department of Neurology, The Second Affiliated Hospital of Guangzhou University of Chinese Medicine, 510405 Guangzhou, China; 2 Department of Neurology, Guangdong Provincial Hospital of Chinese Medicine, 510120 Guangzhou, China; 3 Shenzhen Institute of Advanced Technology, Chinese Academy of Sciences, 518055 Shenzhen, China

**Keywords:** Tanshinone IIA, Brain microvascular endothelial cell, SIRT1, Endoplasmic reticulum stress, Aβ transportation

## Abstract

**Background:**

The disruption of blood-brain barrier (BBB), predominantly made up by brain microvascular endothelial cells (BMECs), is one of the characteristics of Alzheimer’s disease (AD). Thus, improving BMEC function may be beneficial for AD treatment. Tanshinone IIA (Tan IIA) has been proved to ameliorate the cognitive dysfunction of AD. Herein, we explored how Tan IIA affected the function of BMECs in AD.

**Methods:**

Aβ_1–42_-treated brain-derived endothelium cells.3 (bEnd.3 cells) was employed for in vitro experiments. And we performed molecular docking and qPCR to determine the targeting molecule of Tan IIA on Sirtuins family. The APP^swe^/PS^dE9^ (APP/PS1) mice were applied to perform the in vivo experiments. Following the behavioral tests, protein expression was determined through western blot and immunofluorescence. The activities of oxidative stress-related enzymes were analyzed by biochemically kits. Nissl staining and thioflavin T staining were conducted to reflect the neurodegeneration and Aβ deposition respectively.

**Results:**

Molecular docking and qPCR results showed that Tan IIA mainly acted on Sirtuin1 (SIRT1) in Sirtuins family. The inhibitor of SIRT1 (EX527) was employed to further substantiate that Tan IIA could attenuate SIRT1-mediated endoplasmic reticulum stress (ER stress) in BMECs. Behavioral tests suggested that Tan IIA could improve the cognitive deficits in APP/PS1 mice. Tan IIA administration increased SIRT1 expression and alleviated ER stress in APP/PS1 mice. In addition, LRP1 expression was increased and RAGE expression was decreased after Tan IIA administration in both animals and cells.

**Conclusion:**

Tan IIA could promote Aβ transportation by alleviating SIRT1-mediated ER stress in BMECs, which ameliorated cognitive deficits in APP/PS1 mice.

**Supplementary Information:**

The online version contains supplementary material available at 10.1186/s12967-023-03889-y.

## Background

As a progressive neurodegenerative disease in the 21st century, Alzheimer’s disease (AD) has become one of the greatest threats to human health [1]. It is estimated that approximately 50 million people worldwide struggled with AD in 2018, which will increase to 152 million by 2050 [2]. In recent years, more data have emerged that the damage of blood-brain barrier (BBB) is an early pathological hallmark of AD, which occurring before the deposition of Aβ plaques and the appearance of clinical symptoms of cognitive impairment [3]. Brain microvascular endothelial cells (BMECs) are the key components of BBB, along with pericytes, astrocytes, and others [4]. Owing to that tightly sealed endothelial cells and brain capillaries severely restrict the entry of blood-derived molecules into the brain, the transendothelial transportation of Aβ in AD was primarily achieved through specific receptors on BMECs of BBB, such as LRP1 and RAGE [5]. Thus, improving the function of BMECs and promoting Aβ transendothelial transportation are the effective methods for AD treatment.

Tanshinone IIA (Tan IIA), the representative of liposoluble components of *Salvia miltiorrhiza*, has a strong pharmacological effect on blood vessels. Tan IIA plays a therapeutic role in atherosclerosis, myocardial ischemia, coronary heart disease, and cerebral ischemia [6]. In addition, recent studies have demonstrated the neuroprotective properties of Tan IIA against AD [7]. What’s more, it can alleviate neurotoxicity caused by Aβ deposition through anti-inflammation, anti-oxidation, and anti-apoptosis [8]. However, whether Tan IIA could regulate the function of BMECs in AD remained to be explored.

Sirtuins belong to class III histone deacetylase (HDAC), whose catalytic activity depends on the dynamic change of NAD^+^/NADH ratio. Sirtuins is essential for DNA repair, anti-inflammation, and anti-oxidation [9]. In mammals, seven Sirtuins family members have been identified: SIRT1-SIRT7. More and more researches affirm that SIRT1 can modulate multiple AD processes, such as neuroinflammation, oxidative stress, neuronal apoptosis, and neurodegeneration [10]. Decreased SIRT1 level is related with elevated Aβ production in AD patients [11]. Moreover, activation of SIRT1 could regulate the APP processing, such as increasing the expression of ADAM10 and decreasing the expression of BACE1 [12]. SIRT1 can also increase the activity of PGC-1α, which inhibits Aβ production and improves mitochondrial dysfunction [13]. Endoplasmic reticulum stress (ER stress) is a toxic state that occurs when the misfolded proteins accumulated in the ER, leading to the unfolded protein response to restore the protein homeostasis [14]. ER stress-mediated apoptosis results in Aβ peptides neurotoxicity in AD by activating ASK1 and JNK [15]. And ER stress-induced apoptosis can be inhibited by SIRT1 through PI3K-AKT-GSK3β signaling pathway [16]. Additionally, SIRT1 can increase the expression of Cav1 to protect endothelium function by inhibiting endothelial ER stress and miR-204 [17]. Whether Tan IIA could attenuate SIRT1-mediated ER stress and restore the Aβ transportation in BMECs is still unknown.

In the present study, Aβ_1-42_-treated brain-derived endothelium cells0.3 (bEnd0.3 cells) and APP^swe^/PS^dE9^ (APP/PS1) double transgenic mice were employed to unravel the role of Tan IIA in BMECs function and cognitive deficit. Tan IIA postponed the neurodegeneration, and significantly reversed cognitive dysfunction in AD. Tan IIA accelerated Aβ transportation in BMECs through relieving SIRT1-mediated ER stress.

## Methods

### Cell culture

bEnd0.3 cell line was obtained from Jennio Biological Technology Co., Ltd. and incubated by the medium contained 45 mL DMEM (Gibco Invitrogen Corporation), 5 mL FBS (Gibco Invitrogen Corporation), 50 U penicillin, and 50 µg streptomycin at 37 °C with 5% CO_2_ contained in the atmosphere. Once cells had fused to 80–90%, they were treated with 0.25% EDTA for 1.5 min. Complete medium was then added to stop the digestive process. Cells were centrifuged, then the supernatant was discarded, and fresh complete medium was added to re-suspend.

## Cell treatments

**Fig. 1:**
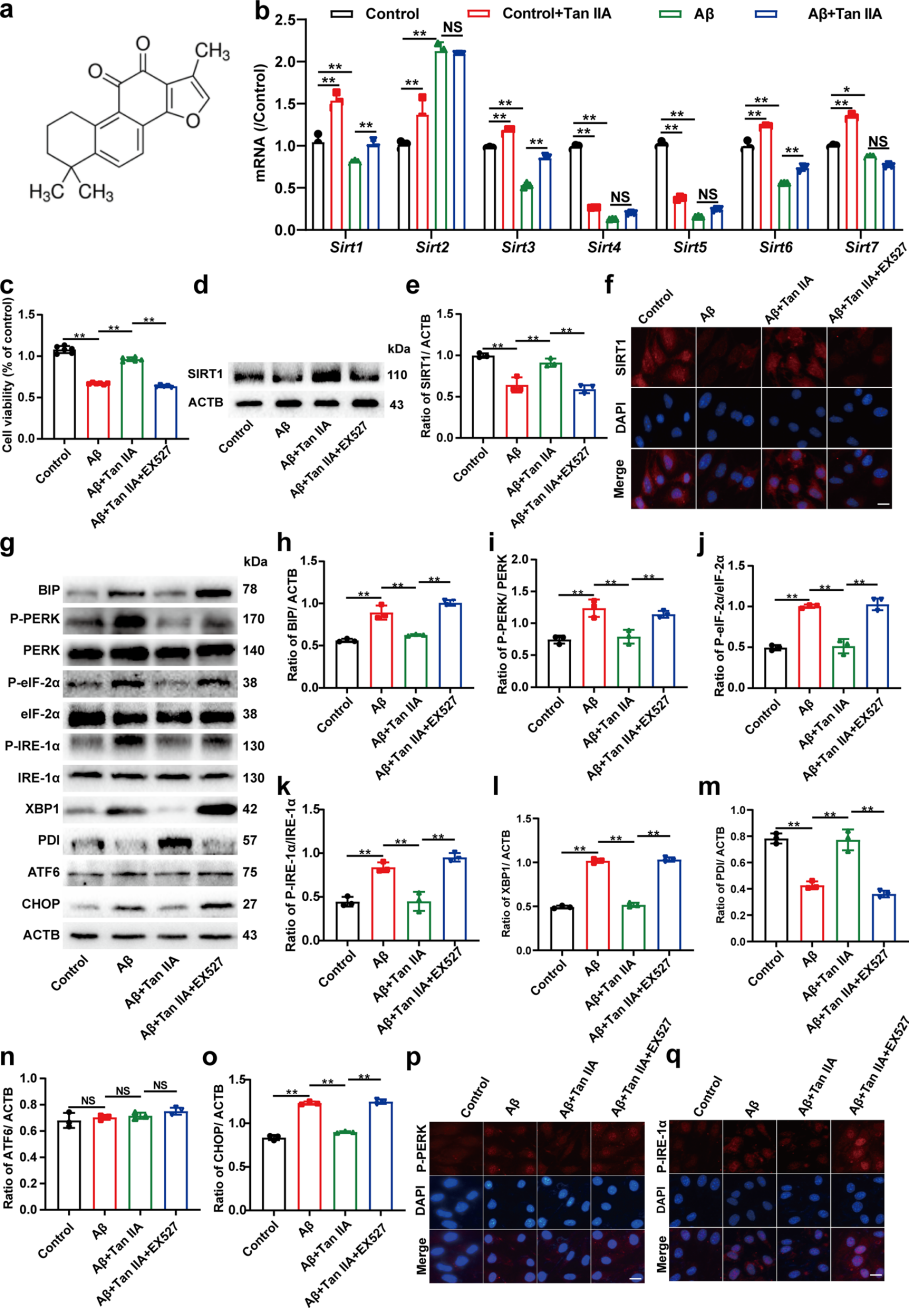
Tan IIA attenuates SIRT1-mediated ER stress in bEnd0.3 cells.** a** Chemical structure of Tan IIA. **b** The mRNA expressions of *SIRT1-7*. **c** bEnd0.3 cells were divided into four groups: control, Aβ (Aβ_1 − 42_ 10 µM), Aβ + Tan IIA (Aβ_1 − 42_ 10 µM + Tan IIA 20 µM), Aβ + Tan IIA + EX527 (Aβ_1 − 42_ 10 µM + Tan IIA 20 µM + EX527 10 µM). Western blot analysis (**d, e**) and indirect immunofluorescence (**f**) of the expression of SIRT1. **g-p** Western blot analysis of ER stress: (**h**) BIP; (**i**) P-PERK/ PERK; (**j**) P-eIF-2/ eIF-2α; (**k**) P-IRE-1α/ IRE-1α; (**l**) XBP1; (**m**) PDI; (**n**) ATF6; and (**o**) CHOP. **p, q** Indirect immunofluorescence of P-PERK (**p**) and P-IRE-1α (**q**). Scale bar is 50 μm. The mRNA expression was first normalized by the housekeeping gene, and then normalized to the controls. The mean ± SD was calculated based on three separate studies, *P < 0.05, **P < 0.01, NS: no significance

Subsequently, the cells were placed in 96-well plates, and administered with different concentration of Tan IIA (99.74%, MedChem Express, Fig. 1a) and Aβ_1–42_. After dissolving Tan IIA with dimethyl sulfoxide (DMSO, Sigma-Aldrich), it was diluted to the appropriate concentrations with DMEM without FBS. And Aβ_1–42_ (GL Biochem Ltd.) was treated with Hexafluoroisopropanol (HFIP, Yuanye Biotechnology) for 1 h at room temperature, fully dissoluted by DMSO, cultivated at 4 °C for 24 h, and then diluted to different concentrations with DMEM.

### Cell viability

The MTT test was used to determine cell viability for establishing the appropriate concentration and time of Tan IIA and Aβ_1–42_ administration. Further experiments were conducted with 20 µM Tan IIA, 10 µM Aβ_1-42_, and 10 µM EX527 (SIRT1 inhibitor, MedChemExpress). Following the pretreatment with Tan IIA for 2 h, cells were incubated with Aβ_1–42_ for another 24 h. bEnd0.3 cells were finally divided into four groups: control, Aβ_1-42_ 10 µM, Aβ_1-42_ 10 µM + Tan IIA 20 µM, Aβ1–42 10 µM + Tan IIA 20 µM + EX527 10 µM.

### Molecular docking

To screen which of the Sirtuins family binds most closely to Tan IIA, molecular docking was used for the preliminary prediction. To begin with, the PDB format files of SIRT1-7 were retrieved and downloaded from RCSB PDB database. Then, these files were processed by AutoDockTools 1.5.6 for dehydration, adding polarity hydrogenation and Gasteiger charges, and saved in PDBQT format. Secondly, the PubChem database was employed to obtain the 3D structure of Tan IIA. Thirdly, the AutoDockTools was employed to create the grid box with the size and coordinates recorded. Afterwards, both the molecular docking was performed and binding energy was calculated by Autodock Vina 1.1.2. Finally, the interaction between SIRT1-7 and Tan IIA were visualized by PyMOL 2.4.0.

### qPCR

A total RNA extract was prepared from bEnd0.3 cells using Trizol reagent. The concentration of RNA was then measured by NanoPhotometer (Implen). Evo M-MLV RT Kit (Accurate Biology) was employed to remove the genomic DNA and perform the reverse transcription. PCR amplification was done using a SYBR green kit (Accurate Biology). After amplification, the melting curve and amplification curve were exported, while the 2 ^−ΔΔCt^ method was used to calculate the relative expression levels of each gene. Table 1 listed the forward and reverse primers utilized in this study.

**Table 1:** Forward and reverse primers utilized for qPCR

Gene	Forward primers (5’–3’)	Reverse primers (5’–3’)
*Sirt1*	CGGCTACCGAGGTCCATATAC	CCGCAAGGCGAGCATAGATA
*Sirt2*	GAGCCGGACCGATTCAGAC	TTCGAGGGTCAGCTCGTCTA
*Sirt3*	GATTCGGATGGCGCTTGAC	TCTCCCACCTGTAACACTCCC
*Sirt4*	TTTATCCCGGCAAAACCCGA	CCGCTCATTCTTATTCTGTCTGG
*Sirt5*	TCCCCACCGCTTTTTGCAG	GCCATATTTGAACTTGGACGAGC
*Sirt6*	GAGTGTGGGGCTGTCAGT	GGGGCCTCTGAAGTCGGG
*Sirt7*	AGGGTCCAGCTTGAAGGTACTA	CTCGACAGCCTCTTCTGTCTC
*β–actin*	CACTGTCGAGTCGCGTCC	TCATCCATGGCGAACTGGTG

### Animals and treatment

The Nanjing University provided APP/PS1 transgenic mice, as well as transgenic negative mice of the same sex and age. At six months old, all mice were then randomly assigned to 4 groups (each with ten mice): WT group, APP/PS1 group, Tan-L group, and Tan-H group. Mice in WT group and APP/PS1 group were administrated by 0.9% saline, while mice in Tan-L group and Tan-H group were treated by Tan IIA at doses of 10 and 20 mg/kg/d. Tan IIA was dissolved in 0.2% DMSO and diluted with 0.9% saline before administrated to the animals. All mice were fed through gavage once a day for 8 weeks. The method of administration to the animals were determined with reference to the previously described protocol [18]. In all experiments involving animals, the guiding principles proclaimed by Guangzhou University of Chinese Medicine (20,200,719,007) were strictly followed.

### Open field test

We performed the open field experiment according to our previous study [19, 20]. The spontaneous motor activity of the APP/PS1 double transgenic mice was measured by open field test through placing them in the center of the open field to exercise freely for 5 min. Next, the central region distance was recorded by SuperMaze software (Xinruan).

### Novel object recognition test

The APP/PS1 transgenic mice also underwent a novel object recognition test to evaluate their episodic memory ability. We performed the novel object recognition test according to our previous study [21, 22]. To begin with, they were provided free access to the non-object area for 10 min one by one on the first day. On the second day, a pair of objects with the same color and shape (object A and A1) was positioned in the area for mice to investigate. 24 h later, a novel object (object B) with different color and shape replaced one of the original objects (object A1) for mice to explore. The time mice spent exploring new and old object (object B and A) were recorded by SuperMaze software (Xinruan) with recognition index calculated.

### Morris water maze test

Based on Morris’s approach, the Morris water maze test measures the memory and learning abilities of APP/PS1 mice [23]. The Morris water maze device consists of a circular pool, a platform hidden 1 cm below the water surface, and a trajectory acquisition system. The first day the mice were taught to find where the platform was, and then the place navigation test was conducted for 5 consecutive days. During this period, the escape latency and navigation paths were recorded by record system of water maze apparatus. On the 7th day, mice were given to participate in space exploration experiment to swim freely with the fixed platform removed. Afterwards, the number of crossing the fixed platform, time spent in the quadrant of hidden platform, and the swimming speed were measured to assess the spatial reference memory.

### ^18^ F-FDG PET/CT

The blood glucose levels of each mouse were monitored before the PET/CT imaging. When the blood glucose level was in the normal range (7.0–10.1 mmol/L), the mouse was approved to perform the ^18^ F-FDG PET/CT scanning. And food and water were withheld from the experimental animals for 6 h prior to the anesthesia. Then the anesthesia was achieved by inhaling pure oxygen mixed with 1.5% isoflurane during the experiment. Next, the radioactive tracer ^18^ F-FDG was injected into the tail vein of mice after full anesthesia. After 1 h of ^18^ F-FDG injection in mice, 10 min-PET images were acquired and statistically evaluated.

### Measurement of catalase (CAT), malondialdehyde (MDA), total superoxide dismutase (T-SOD), and glutathione peroxidase (GSH-PX)

GSH-PX, CAT, and T-SOD are 3 kinds of antioxidant enzymes, and MDA is a lipid peroxide degradation product. One of the distinguishing features of AD is oxidative stress, characterized by a low activity of antioxidant (GSH-PX, CAT, SOD) and high level of MDA production. These oxidative stress markers can reflect the free radical content in tissues. In this study, The Nanjing Jiancheng Bioengineering Institute provided the detection kits for CAT, MDA, T-SOD, and GSH-PX. The supernatant was obtained after homogenizing and centrifuging the samples to determine the protein concentration by the NanoPhotometer. A series of measurements were carried out in line with the manufacturer’s instructions to determine the level of MDA, CAT, T-SOD, and GSH-PX. Finally, the absorbance was determined by Universal Microplate Spectrophotometer (Bio-Rad).

### Western blot

RIPA buffer containing proteinase and phosphatase inhibitors was utilized for homogenizing and lysing bEnd0.3 cells and the cortex tissues, followed by SDS-PAGE with equal amounts of protein separated. Transferring the proteins to PVDF membranes (Millipore) and then blocking them in 5% skim milk for 70 min at room temperature were followed by incubation of the membranes through adding primary antibodies overnight at 4 °C and subsequent secondary antibodies for 90 min at room temperature. Routinely, protein load was determined by Immobilon Western Chemiluminescent HRP Substrate (Millipore) and quantified by NIH software Image J.

### Nissl staining

Paraffin-embedded perfused brain tissues were cut into sections of 5-µm thickness for Nissl staining. After sections had been dewaxed and rehydrated with xylene, ethanol, and distilled water, they were immersed into Nissl stain (Nanjing Jiancheng Bioengineering Institute) for 10 min at room temperature. Subsequently, the slides were washed by distilled water, dehydrated by ethanol, transparentized by xylene, sealed with neutral balsam, and eventually viewed by a light microscope (Leica Microsystems). For the analysis of hippocampal CA2 and cortex regions, Image J was used to count the number of Nissl bodies, and GraphPad Prism was used for the statistical analysis.

### Immunofluorescence

Brain paraffin sections were prepared by embedding the perfused brain tissues in paraffin and then cutting it into 5-µm thick sections for indirect immunofluorescence. After being dewaxed and rehydrated by xylene, ethanol, and distilled water, the slides were heated by microwave in sodium citrate buffer (Biosharp) for antigen retrieval. Cellular immunofluorescence was performed in 24-well plates, with bEnd0.3 cells seeded at 0.25 × 10^6^ per well, treated with Tan IIA, Aβ_1 − 42_, and EX527. After that, the cells were fixed with 4% paraformaldehyde (Aladdin) for 20 min at room temperature. In the next step, we permeabilized the cells and sections with 0.5% Triton X-100 (Solarbio) at 37 °C for 15 min, and then blocked them with 5% Bovine Serum Albumin (BSA, Solarbio) at 37 °C for 30 min, and subsequently incubated them with primary antibodies and secondary antibodies for the same temperature and time as western blot. After being sealed by antifade mounting medium with DAPI (Beyotime), images were obtained under a Leica Microsystems light microscope and analyzed by Image J software (n = 3).

### Thioflavin T staining (Th-T staining)

The brain paraffin sections were dewaxed with xylene, rehydrated with ethanol and distilled water, and washed three times by PBS for 5 min. Then, each 5-µm thick section was incubated with 80 µL Thioflavin T mixture (1 mg Thioflavin T dissolved in 100 µL PBS) for 10 min. Next, the slides were washed by PBS, dehydrated by ethanol, sealed with neutral balsam, and eventually analyzed by fluorescence microscope (Leica Microsystems). Notably, Thioflavin T staining was performed in the cortex and hippocampus of the mice, with three images from three mice analyzed for each treatment.

### Statistical analysis

GraphPad Prism 8.3.0 software (GraphPad) and SPSS 25.0 statistical software (IBM) were employed to perform all statistical analysis. All values were presented in the form of mean ± standard deviation (SD). when comparing differences among more than two groups, one-way ANOVA and two-way ANOVA were applied, followed by Tukey HSD test for multiple comparisons. One-way ANOVA was used for one independent variable, while two-way ANOVA was performed for two independent variables. It was determined that statistical significance was defined as *P*< 0.05.

## Results

### Tan IIA attenuates SIRT1-mediated ER stress in bEnd0.3 cells

Aβ_1–42_ was treated to bEnd0.3 cells with concentrations of 1, 5, 10, and 20 µM for 24 h, and the cell survival rate reached to 93.9%, 78.6%, 51.2%, 45.8% (Additional file 1: Fig. S1a). The Aβ_1-42_ was then chosen for further experiments at a concentration of 10 µM. Tan IIA was treated to bEnd0.3 cells with concentrations of 0.01, 0.1, 1, 10, 20, 50, and 100 µM for 24 h. As shown in Additional file 1: Fig. S1b, the cell viability decreased significantly when Tan IIA concentration reached 50 µM. Also, bEnd0.3 cells were pretreated with various concentrations of Tan IIA (0.01, 0.1, 1, 10, 20 µM) for 2 h before treated with 10 µM Aβ_1–42_ for another 24 h (Additional file 1: Fig. S1c). The MTT result was shown that the maximum effect was achieved at 20 µM. Then, the concentration of 20 µM of Tan IIA was selected for subsequent experiments. To identify the target of Tan IIA acted on, the molecular docking and qPCR analysis were performed. Molecular docking results showed that the binding energy of Tan IIA and SIRT1 was − 10.4 kcal·mol^-1^, higher than that of Tan IIA with other Sirtuins family members (Additional file 1: Fig. S2a–g and Table S1). qPCR results further confirmed that among *Sirt1-7*, Tan IIA could effectively increase *Sirt1* mRNA expression and recover the decreased *Sirt1* expression induced by Aβ_1–42_ (Fig. 1b). Then, EX527, the inhibitor of SIRT1, was used to further explore the role of SIRT1 in the action of Tan IIA. EX527 blocked the protective effect of Tan IIA in Aβ_1–42_-treated bEnd0.3 cells (Fig. 1c). Consistently, western boltting and immunofluorescence results further confirmed that Tan IIA could increase SIRT1 expression in bEnd0.3 cells (Fig. 1d–f and Additional file 1: Fig. S3a).

Subsequently, the relationship between SIRT1 and ER stress was further investigated. There was a marked increase in the protein expressions of BIP (binding protein), P-PERK (phosphorylate PKR-like ER), P-IRE-1α (phosphorylate inositol-requiring enzyme), P-eIF-2α (phosphorylate initiation factor 2), XBP1 (x-box binding protein 1), CHOP (C/EBP homologous protein) and a decrease of PDI (protein disulfide isomerase) in Aβ group as compared to the control group. Tan IIA reversed the expressions of these proteins, while EX527 blocked this effect (Fig. 1g–o). The expression of ATF6 (activating transcription factor 6) did not differ significantly across these groups. Also, the immunofluorescence results of P-PERK and P-IRE-1α exhibited the same trend with western blot (Fig. 1p and q, Additional file 1: Fig. S3b and c). According to these findings, Tan IIA could alleviate SIRT1-mediated ER stress in bEnd0.3 cells, especially through PERK/eIF-2α and IRE-1α/XBP1 pathways.

### Tan IIA up-regulates LRP1 and down-regulates RAGE in bEnd0.3 cells

**Fig. 2:**
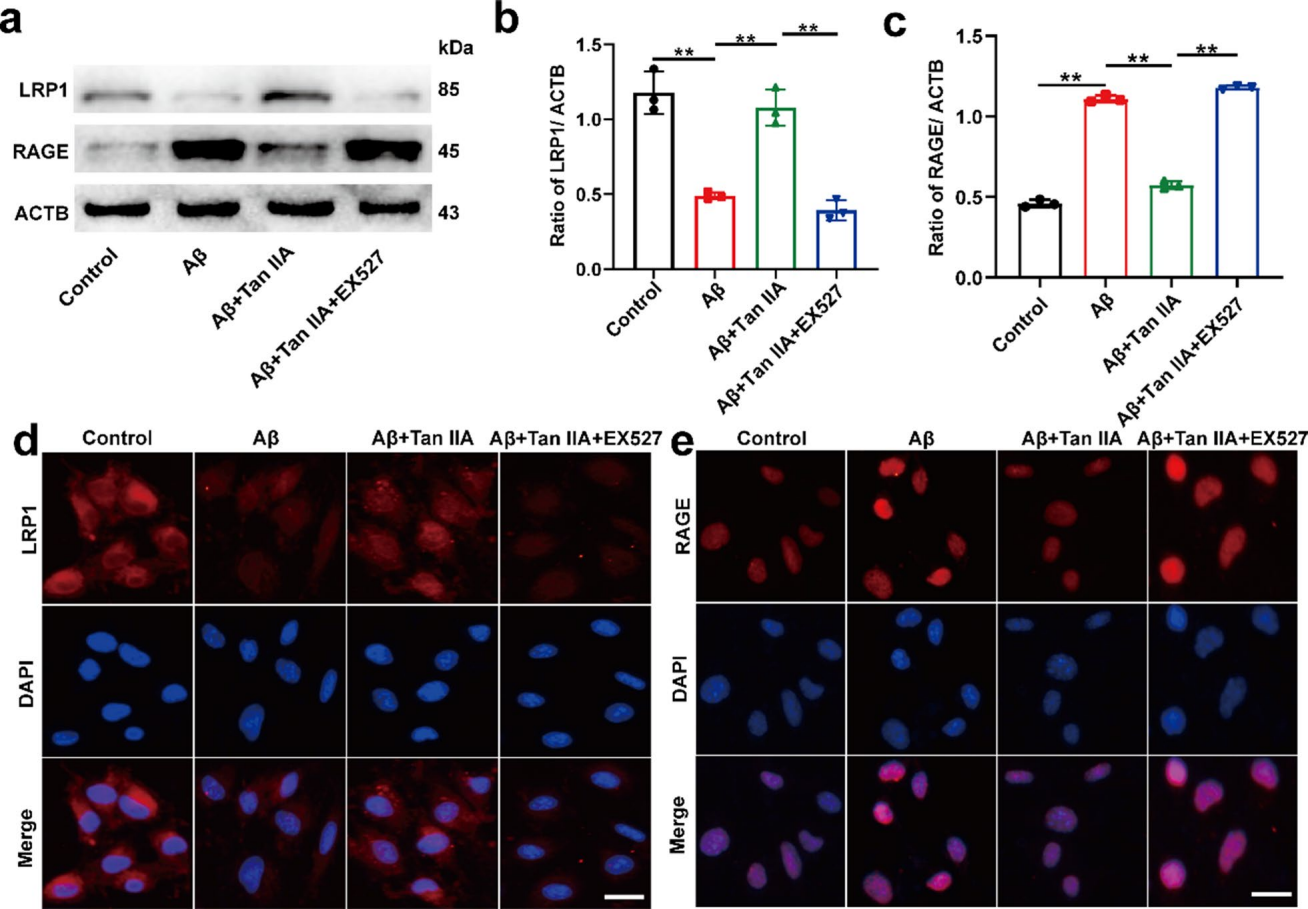
Tan IIA up-regulates LRP1 and down-regulates RAGE in bEnd0.3 cells. **a–c** Western blot analysis of LRP1 (**b**) and RAGE (**c**). **d, e** Indirect immunofluorescence of the expression of LRP1 (**d**) and RAGE (**e**). Scale bar is 50 μm. The mean ± SD was calculated based on three separate studies, *P < 0.05, **P < 0.01, NS: no significance

Western blot (Fig. 2a–c) as well as indirect immunofluorescence (Fig. 2d and e, Additional file 1: Fig. S3d and e) analysis indicated that Aβ_1 − 42_ treatment induced a notable decrease of LRP1 expression and an increase of RAGE expression in bEnd0.3 cells. Tan IIA treatment increased LRP1 expression and decreased RAGE expression. EX527 intervention blocked the effect of Tan IIA. These results suggested that Tan IIA might act on SIRT1 to further mediate Aβ transportation.

### Tan IIA ameliorates cognitive deficits in APP/PS1 mice

**Fig. 3:**
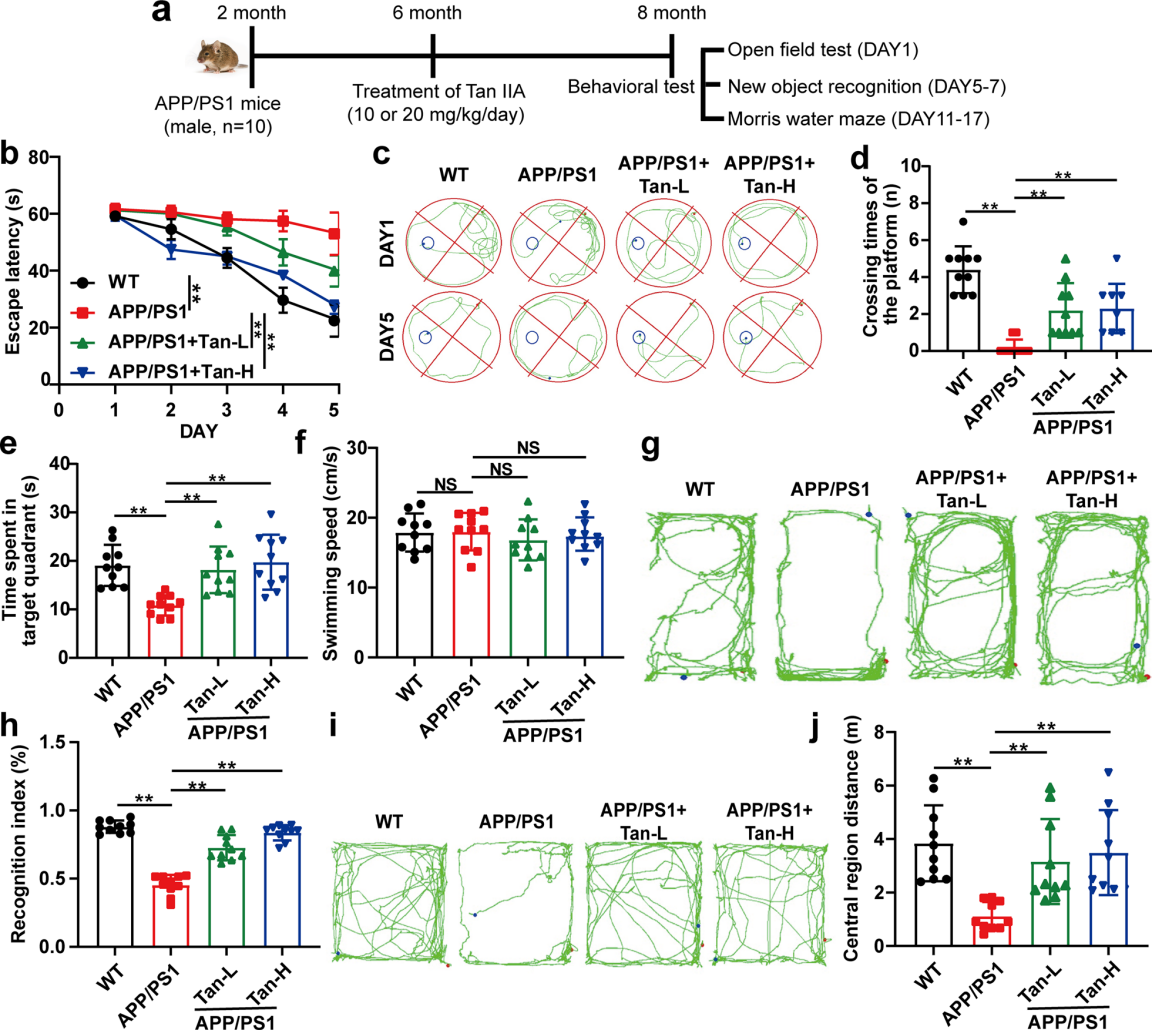
Tan IIA ameliorates cognitive deficits in APP/PS1 mice.** a** Schedule of animal treatments and behavior tests. **b-f** Morris water maze test: **b** Escape latency. **c** Behavioral trajectories. **d** Crossing times in the quadrant of fixed platform. **e** Time spent in the quadrant of fixed platform. **f** The swimming speed. New object recognition test: **g** Behavioral trajectories. **h** The recognition index. Open field test: **i** Behavioral trajectories. **j** The central region distance. Tan-L: Tan IIA (10 mg/kg/d); Tan-H: Tan IIA (20 mg/kg/d). The mean ± SD was calculated based on ten separate studies. *P < 0.05, **P < 0.01, NS: no significance

The therapeutic benefit of Tan IIA for AD was confirmed by in vivo experiments with 10 mice in each group. No toxic side effects were observed after 8-week administration of Tan IIA. As shown in Fig. 3a, the open-field test, novel object recognition test, and Morris water maze test were conducted in order on DAY1, DAY5-7, DAY11-17. There was a three-day interval between each behavioral experiment. The same mouse was subjected to the three behavioral tests. The Morris water maze experiment revealed that the escape latency of the APP/PS1 group was longer than that of the WT group. Tan IIA administration shortened the escape latency of APP/PS1 mice (Fig. 3b and c). Tan IIA administration enhanced the frequency of crossings and time spent in the quadrant of fixed platform (Fig. 3d and e). The swimming speed did not differ significantly across these groups (Fig. 3f). A novel object recognition experiment showed that the less time was cost by APP/PS1 mice to move around new objects than WT mice, while Tan IIA group took more time to explore new objects than APP/PS1 group (Fig. 3g and h). Additionally, APP/PS1 group presented an obvious anxiety-like behavior with shorter central region distance, while Tan IIA relieved the symptom in the open field test (Fig. 3i and j). These results signified that Tan IIA effectively hindered the cognitive deficits of APP/PS1 mice.

#### Tan IIA reduces oxidative stress, alleviates neurodegeneration, and prevents neuronal apoptosis in APP/PS1 mice

**Fig. 4:**
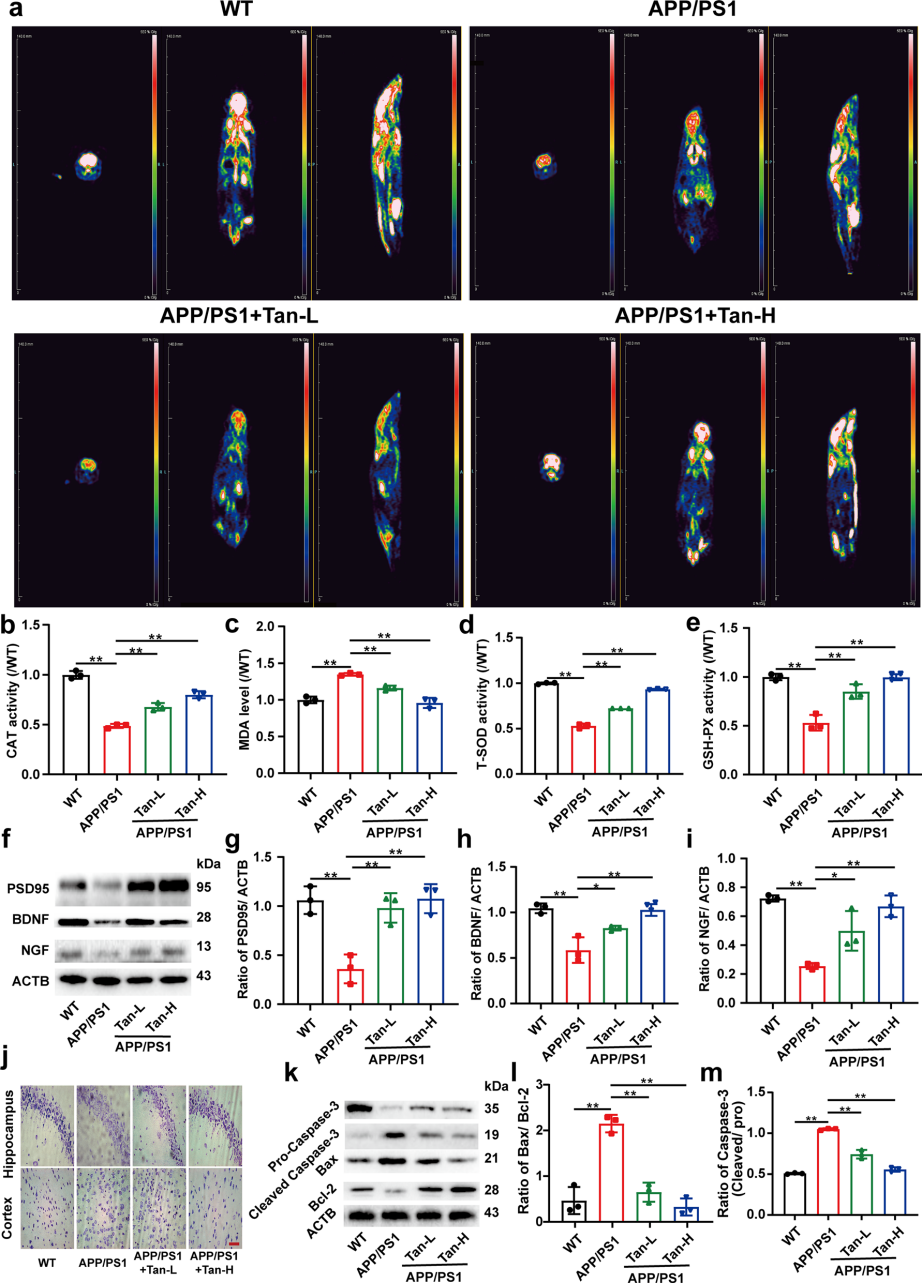
Tan IIA reduces oxidative stress, alleviates neurodegeneration, and prevents neuronal apoptosis in APP/PS1 mice.** a** Axial, sagittal, and axial views of mice were evaluated by ^18^ F-FDG PET/CT. **b-e** The levels of CAT, MDA, T-SOD, and GSH-PX were detected by kits. **f–i** Western blot analysis of PSD95 (**g**), BDNF (**h**), and NGF (**i**). **j** Nissl’s staining in hippocampus and cortex. **k-m** Western blot analysis of Caspase-3 (**l**) and Bax/ Bcl-2 (**m**). Scale bar is 50 μm. Tan-L: Tan IIA (10 mg/kg/d); Tan-H: Tan IIA (20 mg/kg/d). The mean ± SD was calculated based on three separate studies, *P < 0.05, **P < 0.01

Figure 4a showed the glucose uptake of the experimental mice at coronal, sagittal, and axial locations respectively. The glucose uptake was considerably lower in the APP/PS1 group than in the WT group, while improved after Tan IIA administration. In comparison to WT group, the activities of GSH-PX, CAT, and SOD were all decreased significantly, while the level of MDA increased dramatically. Tan IIA protected APP/PS1 mice against oxidative stress (Fig. 4b-e). As shown in Fig. 4f–i, a remarkable decrease of PSD95, BDNF, NGF protein expressions were observed in APP/PS1 group, which were reversed after Tan IIA administration. These results were further proved by Nissl staining. It was evident in the APP/PS1 group that a significantly higher level of neuronal loss was existed, which was alleviated by Tan IIA administration (Fig. 4j, Additional file 1: Fig. S3f and g). In the APP/PS1 group, the expressions of Cleaved Caspase-3 and Bax were markedly elevated, and the expression of Bcl-2 was decreased significantly, whereas Tan IIA administration reduced neuronal apoptosis (Fig. 4k–m). Based on the above results, Tan IIA could inhibit oxidative stress, reduce neurodegeneration and decrease neuronal apoptosis in APP/PS1 mice.

### Tan IIA ameliorates SIRT1-mediated ER stress in the BMECs of APP/PS1 mice

**Fig. 5:**
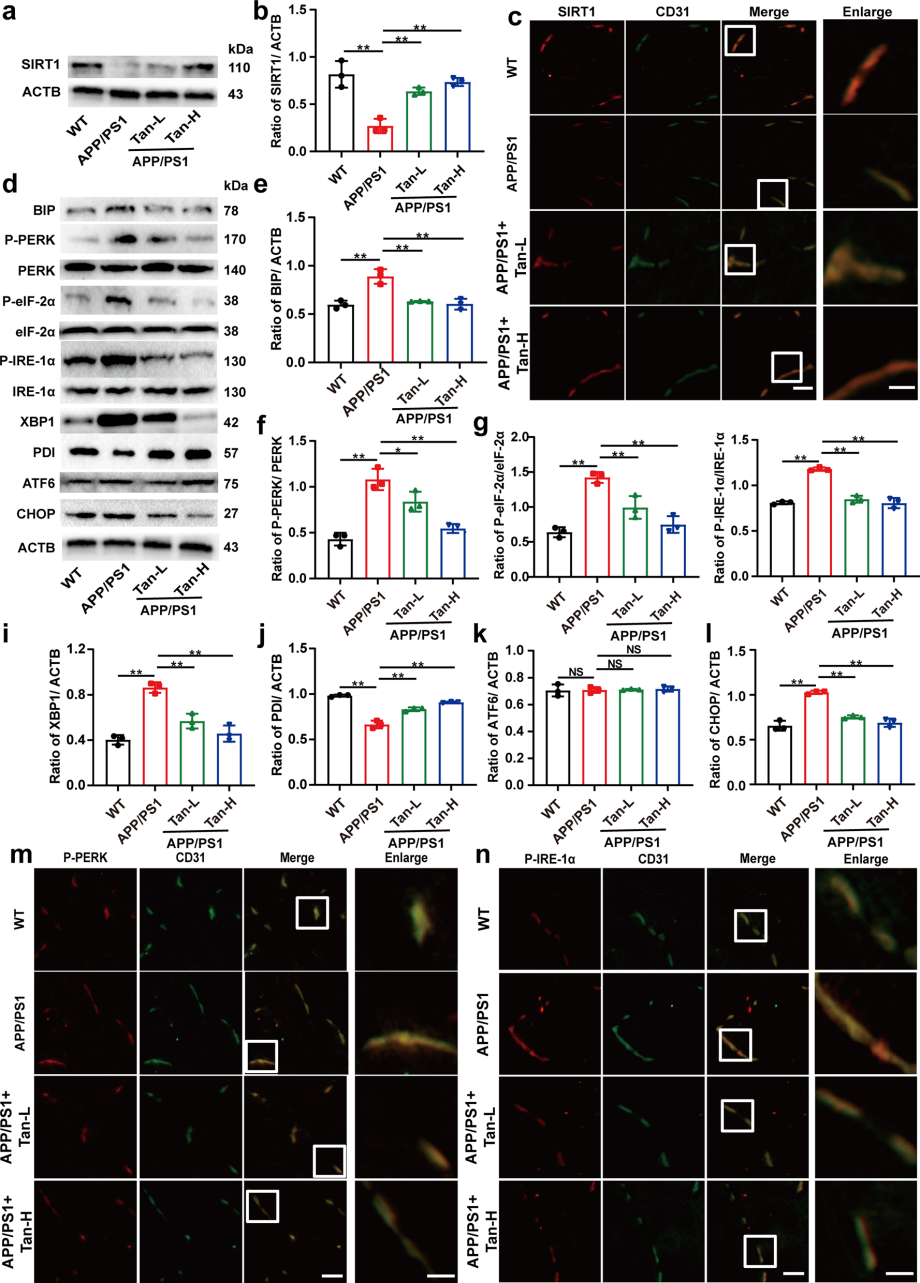
Tan IIA ameliorates SIRT1-mediated ER stress in the BMECs of APP/PS1 mice. **a-c** Western blot (**a, b**) and immunofluorescence (**c**) analysis of the expression of SIRT1. **d-l** Western blot analysis of ER stress in APP/PS1 mice: **e** BIP; **f** P-PERK/ PERK; **g** P-eIF-2α/ eIF-2α; **h** P-IRE-1α/ IRE-1α; **i** XBP1; **j** PDI; **k** ATF6; and **l** CHOP. **m, n** Indirect immunofluorescence of P-PERK (**m**) and P-IRE-1α (**n**) in vascular endothelium of APP/PS1 mice. Scale bar, 50 μm; inset, 20 μm. Tan-L: Tan IIA (10 mg/kg/d); Tan-H: Tan IIA (20 mg/kg/d). The mean ± SD was calculated based on three separate studies. *P < 0.05, **P < 0.01, NS: no significance

Both western blot and immunofluorescence results showed that SIRT1 protein expression was significantly decreased in the BMECs of APP/PS1 mice, whereas Tan IIA treatment elevated SIRT1 expression (Fig. 5a–c, Additional file 1: Fig. S3h). Furthermore, western blot analysis showed Tan IIA treatment ameliorated ER stress in the BMECs from APP/PS1 mice, especially through PERK/eIF-2α and IRE-1α/XBP1 pathways (Fig. 5d–l). Then, the immunofluorescence results of P-PERK and P-IRE-1α presented the same trend as western blot analysis (Fig. 5m and n, Additional file 1: Fig. S3i and j). In these studies, Tan IIA was shown to activate SIRT1 and reduce ER stress in BMECs of APP/PS1 mice.

### Tan IIA promotes Aβ transendothelial transportation in APP/PS1 mice

**Fig. 6:**
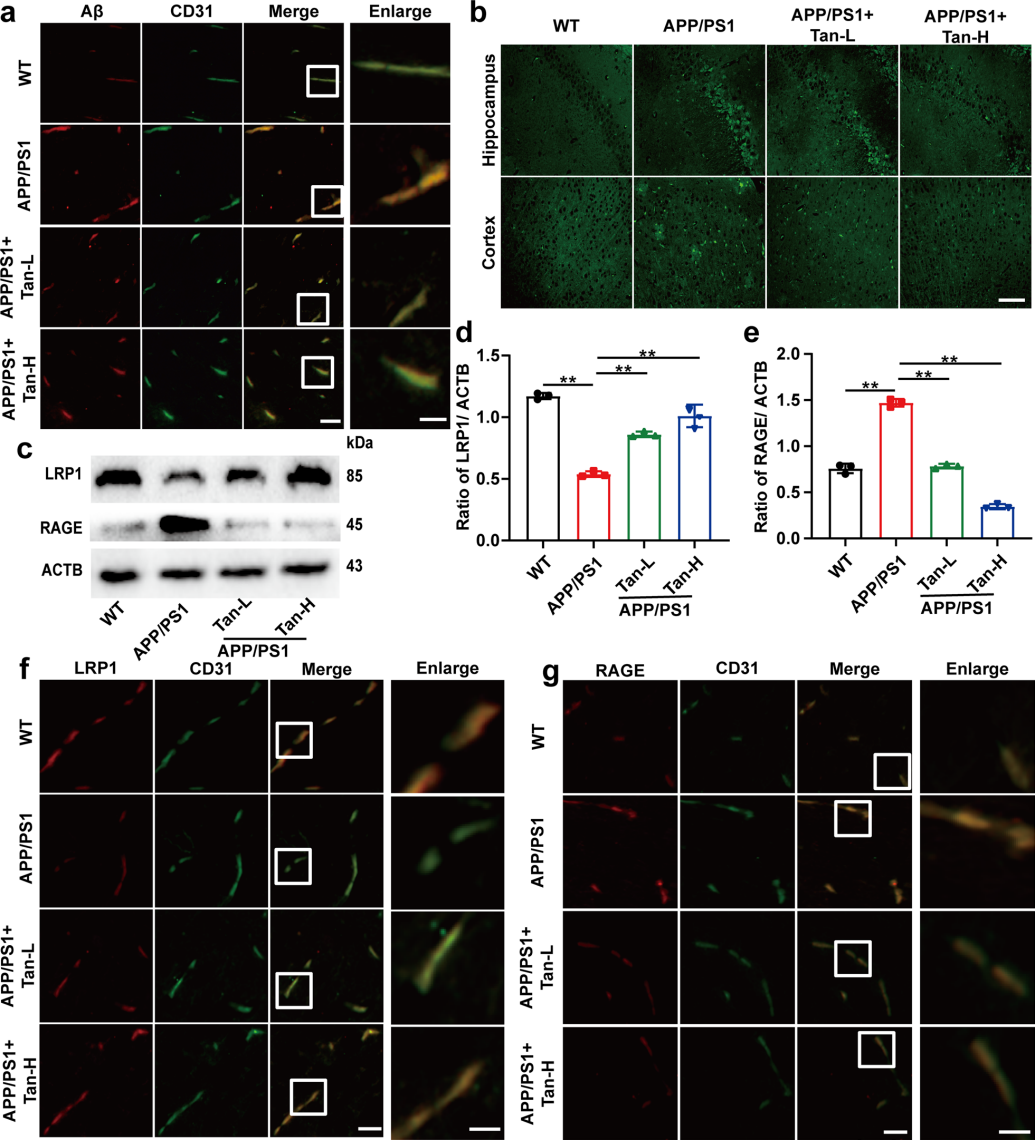
Tan IIA promotes Aβ transendothelial transportation in BMECs of APP/PS1 mice.** a** indirect immunofluorescence of the Aβ aggregation in vascular endothelium of APP/PS1 mice. **b** Thioflavin T staining of the Aβ aggregation in hippocampus and cortex of mice. **c-e** Western blot analysis of LRP1 and RAGE in APP/PS1 mice: **d** LRP1; **e** RAGE. **f, g** Indirect immunofluorescence of LRP1 (**f**) and RAGE (**g**) in the vascular endothelium of APP/PS1 mice. Scale bar, 50 μm; inset, 20 μm. Tan-L: Tan IIA (10 mg/kg/d); Tan-H: Tan IIA (20 mg/kg/d). The mean ± SD was calculated based on three separate studies. *P < 0.05, **P < 0.01

Thioflavin T staining was performed to measure the Aβ deposition. APP/PS1 group had a higher amount of Aβ than WT group, which can be down-regulated by Tan IIA intervention (Fig. 6a, Additional file 1: Fig. S3k). Immunofluorescence result further illustrated that Tan IIA could prevent Aβ deposited in the vascular endothelium of APP/PS1 mice (Fig. 6b). As shown in Fig. 6c-e, western blot demonstrated a decrease in LRP1 expression and a significant increase in RAGE expression in the APP/PS1 mice. Tan IIA intervention promoted Aβ efflux from the brain of APP/PS1 mice. Also, the immunofluorescence presented the same trend as western blot analysis in vascular endothelium of APP/PS1 mice (Fig. 6f and g, Additional file 1: Fig. S3l and m). According to these findings, Tan IIA could protect against cognitive decline by accelerating Aβ transendothelial transportation.

## Discussion

**Fig. 7:**
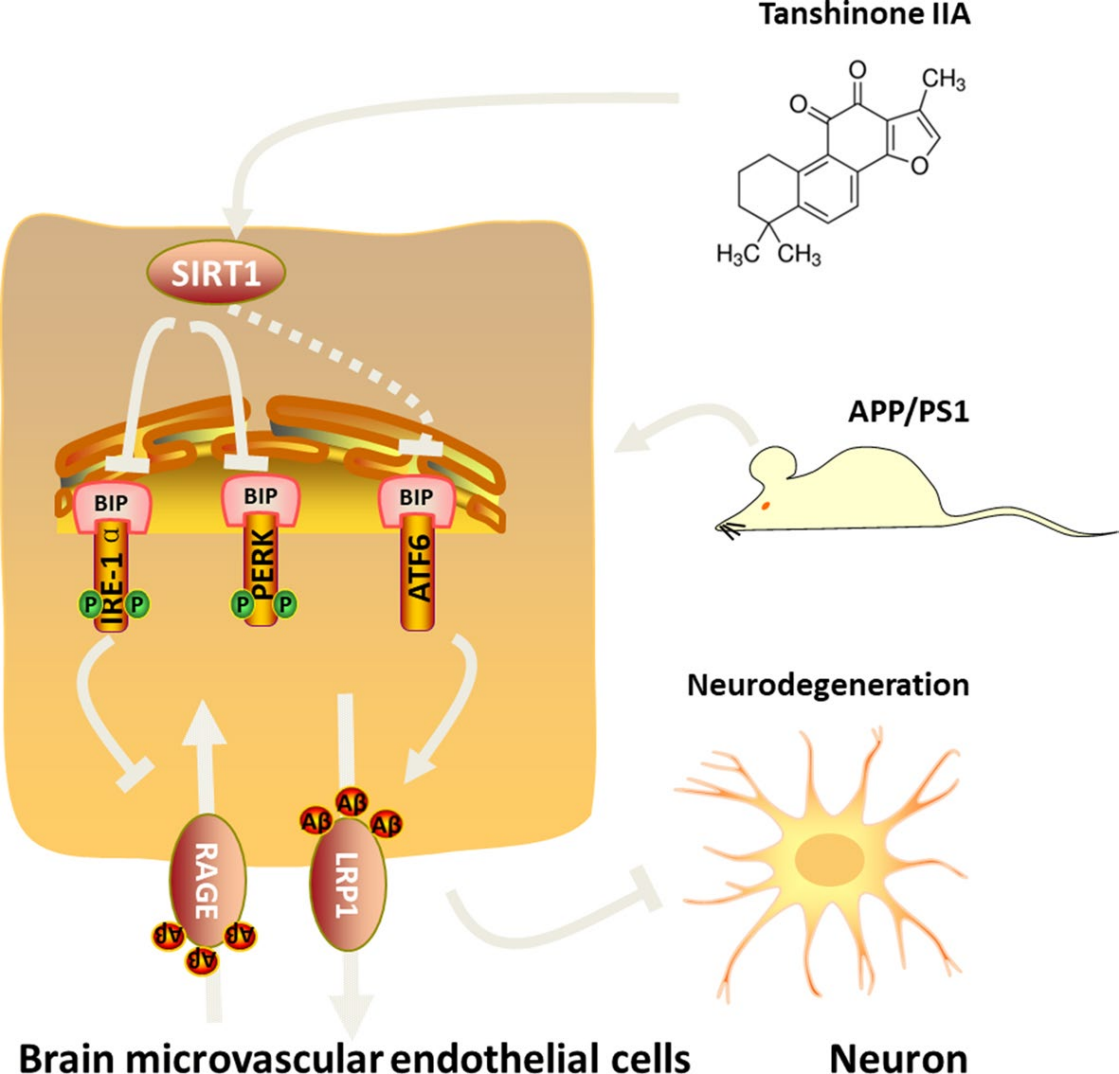
Scheme depicting the mechanism by which Tanshinone IIA (Tan IIA) regulates the function of brain microvascular endothelial cells (BMECs) to improve cognitive deficits in APP/PS1 mice. Tan IIA attenuates SIRT1-mediated endoplasmic reticulum stress and improves Aβ transendothelial transportation by regulating LRP1 and RAGE in BMECs, while it boosts the energy metabolism, reduces oxidative stress, alleviates neurodegeneration, and hinders neuronal apoptosis in neurons

In present study, we uncovered that 8-week administration of Tan IIA could promote Aβ transportation in BMECs through ameliorating SIRT1-mediated ER stress, thereby promoting cognitive recovery in APP/PS1 mice. Meanwhile, Tan IIA alleviated oxidative stress, postponed neuronal degeneration, and inhibited neuronal apoptosis. These experimental evidences together demonstrated the effect and potential mechanism of Tan IIA against AD (Fig. 7).

Aβ plaque deposition was considered to be the key initiator for the occurrence of AD [24]. Consequently, reducing the production of Aβ and promoting degradation and transportation of Aβ may be momentous therapeutic approaches for AD [25]. Such as hyaluronic acid used hydrogel, an effective treatment for AD, which could effectively inhibit Aβ aggregation and cytotoxicity [26]. For Aβ transportation, because of tight cellular junctions in BBB, Aβ transendothelial transportation must rely on specialized endothelial transport systems in BMECs, composed of LRP1, RAGE, GLUT1, P-gp, etc. [27]. Among them, the LRP1/RAGE receptor system is important, namely Aβ plaque is transported out of the brain through LRP1, and into the brain through RAGE [28]. APP/PS1 mice exhibit lower expression of LRP1 and higher expression of RAGE, resulting in Aβ deposition in the BBB and further damage of BMECs [29]. Therefore, restoring the function of BMECs and accelerating the Aβ transendothelial transportation are conducive to the treatment of AD.

Tan IIA, the primary active ingredient of *Salvia miltiorrhiza*, has been proved to be capable of improving the function of vascular endothelium through inhibiting extracellular Ca^2+^ inflow and intracellular Ca^2+^ outflow, stimulating the estrogen receptor signaling pathway, and regulating the expression of endothelium-dependent related factors [30]. In addition, Tan IIA has been proven in numerous recent studies to be capable to reverse memory and learning deficits in APP/PS1 mice, which is consistent with our results [7]. More importantly, sodium tanshinone IIA sulfonate (STS) is a constituent of Tan IIA that has been demonstrated to be crucial in Aβ metabolism, such as up-regulating ADAM10, IDE, and NEP expression, and inhibiting BACE1 [31, 32]. Our study corroborated that Tan IIA could promote Aβ transportation in both BMECs from APP/PS1 mice and bEnd0.3 cells.

SIRT1 is a kind of deacetylase belonging to the Sirtuins family, which plays a vital role in cell differentiation, cell senescence and stress response [33, 34]. Increasing evidences suggest that SIRT1 is involved in regulating multiple processes in AD progression, such as anti-neuroinflammation, anti-neurodegeneration, and improving the mitochondrial dysfunction [35, 36]. Also, SIRT1 can improve cognitive dysfunction through repairing DNA damage, degrading toxic proteins, and maintaining synaptic plasticity [37]. Furthermore, SIRT1 participates in Aβ generation by up-regulating ADAM10 and down-regulating BACE1 through cAMP/PKA pathway [12]. Also, SIRT1 plays a vital role in Aβ degeneration by enhancing the expression of NEP to relieve the cadmium-induced ferroptosis and apoptosis [38]. In addition, SIRT1 can be regulated by Tan IIA to inhibit the inflammatory response, to relieve the myocardial ischemia reperfusion injury, and to treat mast cell-mediated allergic diseases [39, 40]. In our study, SIRT1 was found to be activated by Tan IIA to accelerate Aβ transportation in cerebrovascular endothelium through the LRP1/RAGE transport system.

ER stress is a process in which cells activate unfolded protein response (UPR) to correct the misfolding of ER lumen and dysregulation of calcium homeostasis [41]. In the onset and progression of AD, the accumulation and aggregation of Aβ plaque lead to intense imbalance of ER calcium homeostasis, protein misfolding, and ER stress [42]. Sharma et al. found that inhibition of PERK expression in hippocampal CA1 region of adult male mice strengthened neuronal excitability and improved cognitive impairment [43]. Devi et al. illustrated that phosphorylation of eIF2α, mediated by PERK, induced BACE1 elevation and neurodegeneration in APP/PS1 mice [44]. Also, ADAM10 can be up-regulated through IRE-1α/XBP1 Pathway, thus slackening the production of Aβ [45]. Liu et al. uncovered that restraining the P-PERK and P-IRE-1α expressions could increase the expressions of IDE and NEP in Aβ_1–42_-induced HT22 cells [32]. Above all, SIRT1 is a predominant regulator of ER stress, which can markedly reduce UPR and expression of chaperone protein BIP [46]. Also, it can alleviate ER stress by regulating the deacetylation of eIF-2α [47]. Our study verified that activation of SIRT1 reduced P-PERK and P-IRE-1α expressions, while partly blocked by SIRT1 inhibitor (EX527). This manifested that ER stress can be mediated by SIRT1 through PERK/eIF-2α and IRE-1α/XBP1 signaling pathways.

Oxidative stress, the common denominator of neurodegenerative diseases, produces free radicals that can cause oxidative damage to Aβ itself, and then lead to the deposition of Aβ, which is closely related to the pathogenesis of AD [48]. Conversely, Aβ deposition may result in disruption of oxidative balance through mitochondrial dysfunction and lipid peroxidation [49]. Besides, oxidative stress takes negative effects on the structure of neuronal cells, especially mitochondria, triggering mtDNA damage, destroying Ca^2+^ homeostasis, and reducing ATP production, giving rise to neuronal apoptosis [50]. In addition, oxidative stress has been found to be an early precursor to apoptosis and interacts with mitochondria/lysosomal to produce cytotoxicity [51–53]. Furthermore, neurons from APP/PS1 mice exhibit an increased response to apoptosis stimulation due to the production of APP and PS1 [54]. Oxidative stress and neuronal apoptosis gradually induce neurodegeneration, which can be reflected by the Nissl staining results [55, 56]. In this study, Tan IIA successfully inhibited oxidative stress, neuronal apoptosis, and neurodegeneration in APP/PS1 mice.

## Conclusion

Taken together, our study elucidated that Tan IIA could reverse learning and memory decline by facilitating Aβ transportation, and its possible mechanism was to relieve SIRT1-mediated ER stress in the brain microvascular endothelium cells. However, the detailed mechanism behind SIRT1 mediating ER stress (such as whether its deacetylase function contributes to this) remains to be elucidated. Additionally, the neuroprotective effect of Tan IIA on AD still needs to be confirmed in clinical trials. The pharmacological properties of Tan IIA, which is derived from *Salvia miltiorrhiza*, drive it to be a promising drug for treating AD.

## Supplementary Information

**Additional file 1.** Additional figures and table.

## Data Availability

All data or resources used in the paper are available by reasonable requirements to the correspondence authors.
