# Supplementary material for: Tanshinone IIA ameliorates Aβ transendothelial transportation through SIRT1-mediated endoplasmic reticulum stress
Source: J Transl Med. 2023 Jan 20;21:34. doi: 10.1186/s12967-023-03889-y (PMC9854034; doi:10.1186/s12967-023-03889-y)
Supplement: Supplementary file 1 — Additional file 1. Additional figures and table. [file 12967_2023_3889_MOESM1_ESM.docx]

**Table S1 Molecular docking of Sirtuins family and Tan-ⅡA**

| Protein | PDB ID | Grid center | | | Docking score（kcal·mol^−1^) |
| --- | --- | --- | --- | --- | --- |
|  |  | x | y | z |  |
| SIRT1 | 4ZZI | 7 | 47 | -4 | -10.4 |
| SIRT2 | 4RMH | 15 | 20 | 5 | -7.7 |
| SIRT3 | 4BN4 | 16 | 7 | 2 | -8.9 |
| SIRT4 | 5OJ7 | 18 | 18 | 48 | -9.2 |
| SIRT5 | 6EQS | 6 | 32 | 75 | -9.8 |
| SIRT6 | 6XV6 | -56 | -53 | -44 | -9.0 |
| SIRT7 | 6G0S | 32 | 9 | 30 | -7.2 |


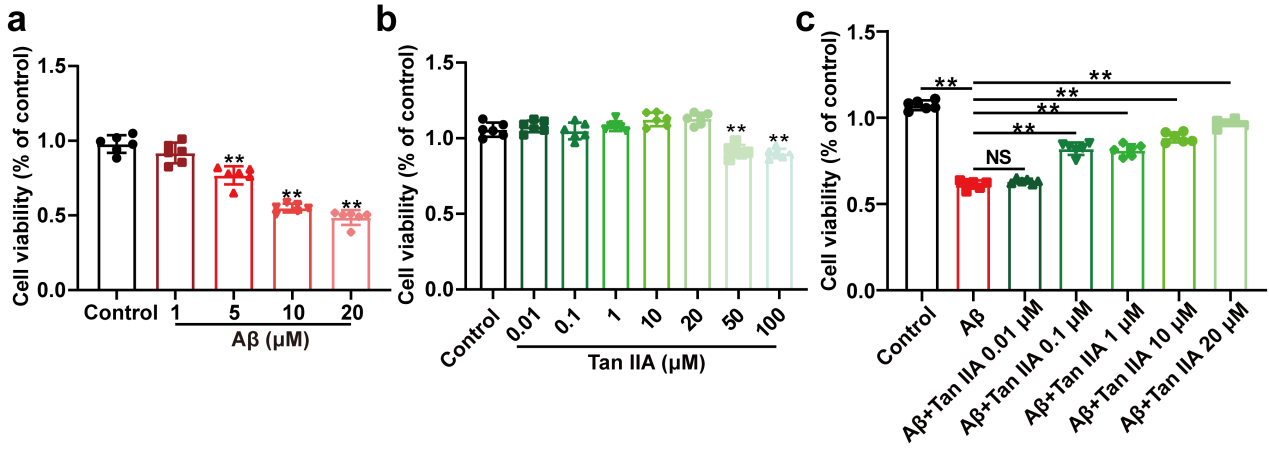


**Fig. S1 a** Aβ_1–42_ was treated to bEnd.3 cells with concentrations of 1, 5, 10, and 20 μM for 24 h. **b** Tan IIA was treated to bEnd.3 cells with concentrations of 0.01, 0.1, 1, 10, 20, 50, and 100 μM for 24 h. **c** bEnd.3 cells were pretreated with various concentrations of Tan IIA (0.01, 0.1, 1, 10, 20 μM) for 2 h before being cultured with 10 μM Aβ_1–42_ for another 24 h. The MTT assay was used to assess the cell viability. The mean±SD was calculated based on six separate studies. **P* < 0.05, ***P* < 0.01, NS: no significance.

**
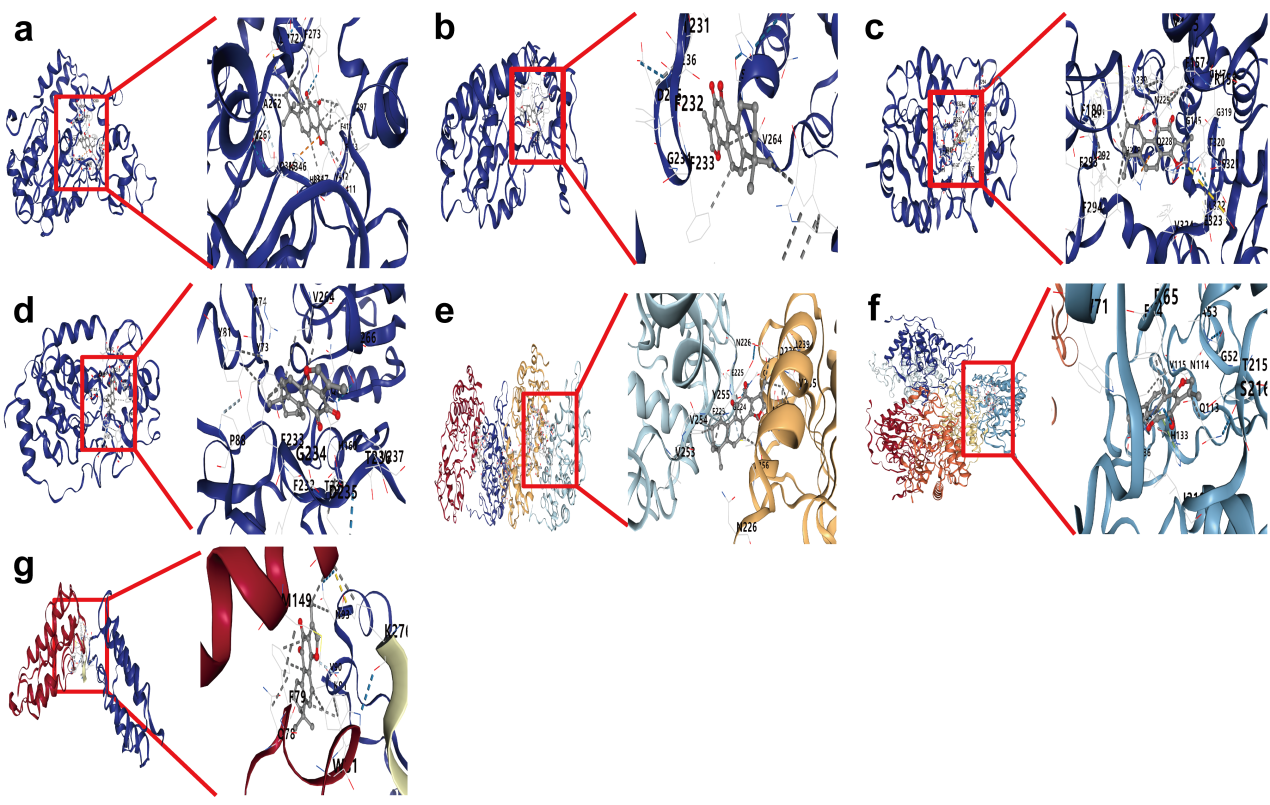
**

**Fig. S2** Molecular docking of Sirtuins family and Tan ⅡA. **a** SIRT1. **b** SIRT2. **c** SIRT3. **d** SIRT4. **e** SIRT5. **f** SIRT6. **g** SIRT7.

**
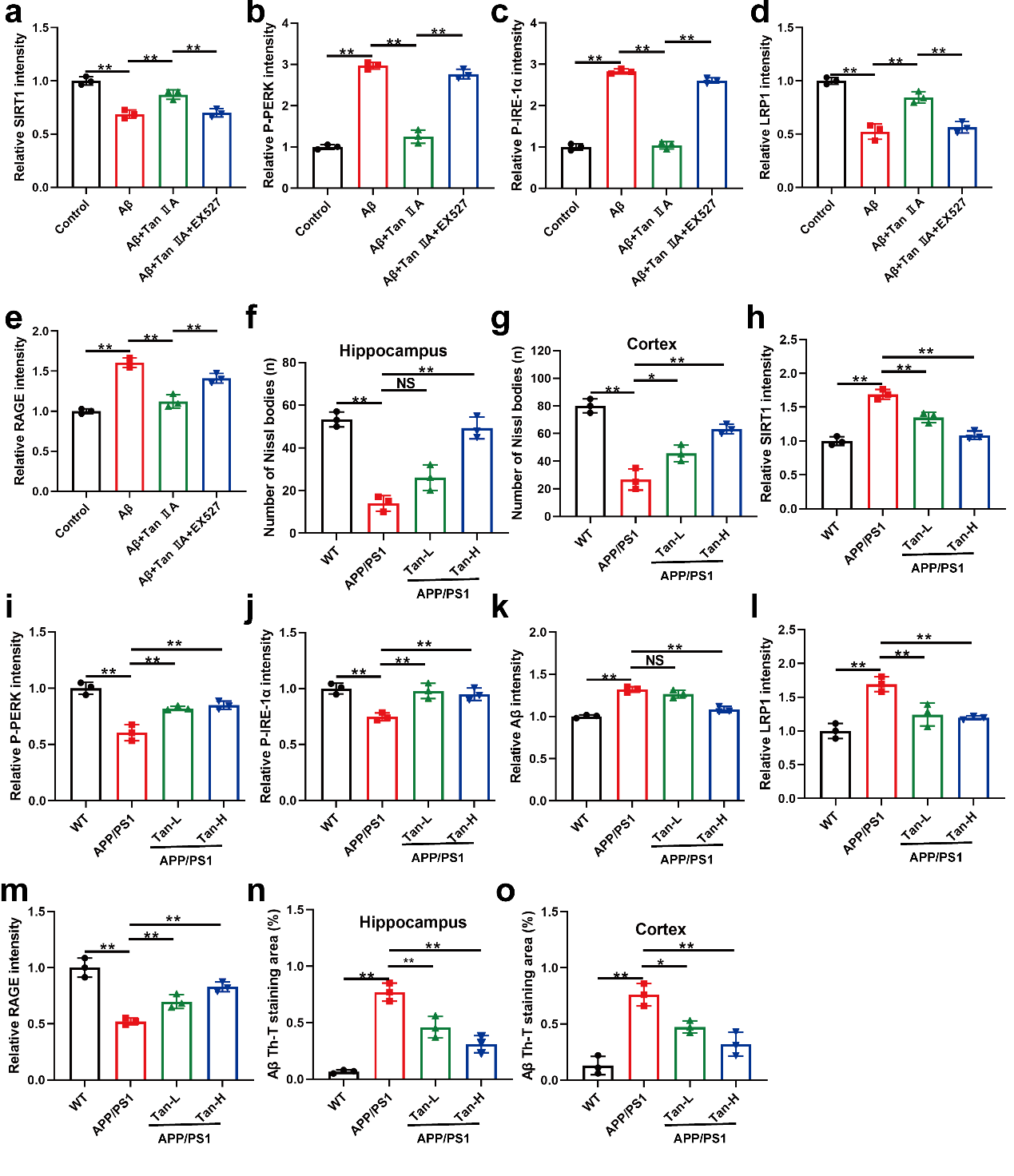
Fig. S3** Quantification of the immunostaining, Nissl’s staining and Th-T staining results. (a-e) Immunostaining results of SIRT1, P-PERK, P-IRE-1α, LRP1, RAGE in bEnd.3 cells. (f-g) Nissl’s staining results of hippocampus and cortex. (h-m) Immunostaining results of SIRT1, P-PERK, P-IRE-1α, Aβ, LRP1, RAGE in the vascular endothelium of APP/PS1 mice. (n-o) Aβ Th-T staining area in the hippocampus and cortex.
